# Effect of maternal *Helicobacter Pylori* infection on gestational weight gain in an urban community of Uganda

**DOI:** 10.11604/pamj.2017.28.145.9989

**Published:** 2017-10-16

**Authors:** Ronald Wanyama, Gerald Obai, Pancras Odongo, Michael Kagawa, Rhona Baingana

**Affiliations:** 1 Department of Biochemistry, Faculty of Medicine, Gulu University, Gulu, Uganda; 2 Department of Physiology, Faculty of Medicine, Gulu University, Gulu, Uganda; 3 Department of Internal Medicine, Faculty of Medicine, Gulu University, Gulu, Uganda; 4 Department of Obstetrics & Gynecology, College of Health Sciences, Makerere University, Kampala, Uganda; 5 Department of Biochemistry and Sports Science, School of Biolsciences, Makerere University, Kampala, Uganda

**Keywords:** H. pylori infection, pregnancy, gestational weight gain, parity, Kampala, Uganda

## Abstract

**Introduction:**

Maternal *Helicobacter pylori (H. pylori)* infection has been associated with undesirable effects during pregnancy such as; hyperemesis gravidarum, anemia, intrauterine fetal growth restriction and miscarriage. Our aim was to document the effect of *H. pylori* infection on gestational weight gain (GWG) in a low-income urban setting in Uganda.

**Methods:**

This was a prospective cohort study conducted in Kampala between May 2012 and May 2013. The participants were HIV negative, *H. pylori* positive and *H. pylori* negative primigravidae and secundigravidae. Recruitment was at gestation age of eighteen or less weeks and follow up assessments were carried out at 26 and 36 weeks gestation age. *H. pylori* infection was determined using *H. pylori* stool antigen test. Maternal weight and height were measured, and body mass index (BMI) and rates of GWG were calculated.

**Results:**

The participants’ mean±standard deviation (sd) age was 20.9±2.7 years. Primigravidae were 68.8% (n = 132) and 57.3% (n = 110) of the participants were positive for *H. pylori* infection. Low pre-women pregnancy BMI (< 18.5 kg/m^2^) was recorded in 14.6% (n = 28). The mean±sd rate of GWG during second and third trimesters was 300.5±79.7 grams/week. The mean±sd weight gained by 36 weeks of gestation was 9.6±2.2 kg while gestation age at delivery was 39.4±1.0 weeks. Factors independently associated with the rates of GWG during the second and third trimesters were parity (P=0.023), *H. pylori* infection (P = 0.006), pre-pregnancy BMI (P = 0.037), height (P = 0.022) and household income (P = 0.003).

**Conclusion:**

*H. pylori* infection is associated with low rates of GWG among primigravidae and secundigravidae.

## Introduction

*Helicobacter pylori* (*H. pylori*) infection affects approximately one half of the world population and it is more prevalent in developing countries [1, 2]. This microorganism colonizes the stomach. Typically, it is acquired during childhood and causes asymptomatic chronic infection [3]. However, pregnancy increases the susceptibility to *H. pylori* infection [4] probably due to decreased cell-mediated cytotoxic immune response [5]. Although many infected individuals are asymptomatic, *H. pylori* is an important health problem. *H. pylori* infection has been recognized as a major cause of various gastroduodenal diseases, such as chronic gastritis, peptic ulcer disease, and gastric cancer [3]. In Uganda the prevalence of *H. pylori* infection in dyspepsia patients who underwent endoscopy was 74% and 86% in patients with cancer and benign tumors [6, 7]. Recently, Baingana et al., found the prevalence of *H. pylori* infection of 60.5% among pregnant women attending an antenatal clinic in Kampala [8]. Pregnancy is a physiological condition in which a marked increase in body weight occurs over a short period of time. An optimum weight gain over the course of pregnancy, as recommended by the Institute of Medicine (IOM), is one that produces a healthy newborn [9]. Optimum weight gain also provides sufficient postpartum maternal fat stores to support lactation without increasing obesity risk [9]. Furthermore, there is evidence to show that maternal pre-pregnancy weight and the weight gained during pregnancy influence birth weight [10]. However, gestational weight gains below the IOM recommendation are common in developing countries [11]. Inadequate gestational weight gain increases the risk of preterm delivery and low birth weight infants [12, 13]. Current evidence shows that total gestational weight gain and rate of weight gain decreases with increasing pre-pregnancy body mass index (BMI) [14] and this is in agreement with 2009 IOM recommendations [9].

*H. pylori* infection in pregnancy is associated with many adverse effects, such as extreme, persistent nausea and vomiting (hyperemesis gravidarum) [15, 16], neural tube defects in newborns, pre-eclampsia, intrauterine fetal growth restriction and miscarriage, and thrombocytopenia [17-20]. Conditions such as nausea and vomiting reduce appetite. This can lead to reduced food intake and in due course, inadequate supply of nutrients to the body. Furthermore, *H. pylori* infection has been associated with reduced production of ghrelin and increased levels of gastric leptin [21, 22]. Ghrelin increases appetite, facilitates fat storage, and may influence energy homeostasis [23-25]. Increased expressions of gastric leptin make the affected individuals to experience decreased appetites and subsequently weight loss [26]. An association between *H. pylori* and weight loss has been suggested [25, 26]. However, there is limited data on association between *H. pylori* and gestation weight gain especially in developing countries where inadequate GWG is already common. The objective of this study was to establish the association between *H. pylori* infection and maternal weight gain during pregnancy.

## Methods

The study protocol was reviewed and approved by the Research and Ethics Committee of the School of Medicine, Makerere University. Clearance to conduct this study was obtained from Uganda National Council for Science and Technology. Permission to conduct the study at Kawempe Health Centre IV was granted by the Kampala Capital City Authority, Health Department. Participation in the study was voluntary and each participant signed a consent form.

### Study design, site and population

This was a prospective cohort study conducted between May 2012 and May 2013. Pregnant women were followed from early second trimester to late third trimester. The study was conducted at the antenatal clinic of Kawempe Health Centre IV. The Health Centre is supported by the Ministry of Health, Uganda and the services in the antenatal clinic are free to the public. This clinic serves a densely-populated, low-income area in Kawempe Division, one of the five divisions forming Kampala District in Uganda. The division is located in the Northern part of Kampala District. The study targeted HIV negative primigravidae and secundigravidae.

### Sample size

We used the online openEpi software, based on Kelsey Lesley formula (1996) to calculate the sample size. In the formula we used a confidence level of 95%, power of 80%, ratio of *H. pylori* positive to *H. pylori* negative of one. Furthermore, in the formula we used 18 and 35 as the percentages of unexposed and exposed participants with outcome of interest according to Elsick [21]. The exposed group comprised of those who tested positive for *H. pylori* infection while the unexposed group comprised those who tested negative for *H. pylori* infection.

### Recruitment and follow up

A consecutive sampling procedure was used to select participants who met the selection criteria until the sample size was achieved. The participants were chosen as they got registered at the antenatal clinic. Written informed consent for each eligible participant was sought after clear information being given about the study objectives, procedures and benefits. In Uganda HIV testing for pregnant women is recommended and is always done on the day of the first visit to the antenatal clinic. The study participants were recruited as informed volunteers at 12-18 weeks of gestation based on the reported last menstrual period and the experienced midwife’s examination. Follow up assessments were carried out at 26 and 36 weeks of gestation. The study participants were included in our cohort based on the following criteria; between 18-35 years of age, pregnant for the first or second time, HIV negative, carrying a singleton pregnancy, free of any systemic illness such as hypertension, active peptic ulcers, diabetes mellitus or genetic abnormality like sickle cell disease, and between gestation weeks 12-18 at the time of recruitment. However, some of the pregnant women were excluded from this study based on the following criteria; not able to recall their pre-pregnancy weight, not able to schedule their return visits, not able to speak and/or hear, mentally ill, history of drug or alcohol abuse Based on the set exclusion criteria, a total of 56 women were excluded from this study. Fourteen of them could not adhere to the scheduled return visits, two had sickle cell disease, four had alcohol related problems, twenty-eight could not recall their pre-pregnancy weight, six had active peptic ulcers and two were carrying twins.

### Data collection and determination of nutritional status

During the participant's interview, demographic data including social, behavioral and medical history were collected in researcher-administered structured questionnaires. Nutritional status of each participant was assessed using anthropometric parameters. Anthropometric measurements were carried out with the help of a midwife in a closed room when the participant was barefoot and wearing light clothing with the help of a midwife. Body weight was measured using an adult portable beam scale with 150 kg capacity divided into 0.5kg increments (Gmbh & co.kg, Germany model 7621019009). Height was determined with the individual barefoot and in an orthostatic position with the aid of a portable stadiometer consisting of a non-extendable 2 meter measuring tape divided into 0.1cm increments. Body weight and height were measured twice for every participant and the average of the readings was considered as the participant’s weight and height respectively. Each participant’s BMI was calculated using the following formula: BMI = pre-pregnancy body weight (kg) divided by height (m) squared. The BMI was categorized using the World Health Organization criteria as follows; underweight (< 18.5 kg/m^2^), normal weight (18.5-24.9 kg/m^2^), overweight (25.0-29.9 kg/m^2^), obese (≥ 30.0 kg/m^2^) [27]. Pre-pregnancy weight in kg (*W_p_*) considered in this study was that reported by the participant at recruitment. The measured gestation weight at each time point (GWR = weight at recruitment, GW*_26_* = weight at 26 weeks of gestation and GW36 = weight at 36 weeks of gestation) were recorded. Rate of GWG during second trimester was calculated as [(GW26 – GWR)/(26 – gestation age at recruitment)]kg/week. Similarly, rate of GWG during third trimester was calculated as [(GW36 – GW26)/10]kg/week. GWG by 36 weeks was got by subtracting *W_p_* from *W_36_*.

### Stool collection and testing for H. pylori infection

After clear instructions on how to collect the stool sample, each participant was given clean tissue paper on which to deposit the stool. After, she had to immediately transfer a stool sample into stool collection bottle using the scooper which was part of the bottle top. This was done in the antenatal clinic toilet. Stool samples were immediately placed in a cool box with ice packs. The samples were transported everyday from Kawempe Health Centre to the laboratory (~3 km) and stored in a -20°C freezer until analysis was carried out. *H. pylori* stool antigen test, i-Chek cassettes (Chem-Labs Limited, Nairobi, Kenya) were used to analyze the stool samples. It is a rapid one-step chromatographic immunoassay that utilizes a combination of anti-*H. pylori* antibodies and anti-mouse IgG. Instructions given by the manufacturer were followed. Approximately 100 μl of stool of completely thawed stool was brought into the sample diluent tube and vortexed for fifteen seconds. Three drops of the diluted sample were applied to the test and the result was read after fifteen minutes. The results were reported as positive or negative on the basis of the manufacturer's guidelines. A procedural control was included with each test.

### Data analysis

Data were analyzed using Statistical Package for Social Sciences (SPSS) V.15.0 (SPSS Inc., Chicago, IL, USA). Social, demographic and measurement parameters were summarized into frequencies and mean ± standard deviation (sd). The outcome variable was rates of GWG while the independent variables were *H. pylori* infection, pre-pregnancy weight, pre-pregnancy BMI, parity and maternal height. Continuous data were checked for normality. Tests for the significance of association were made using the Pearson chi-square (χ2) test for categorical variables and independent sample *t* test for continuous variables. Factors associated with GWG were determined with linear regression. Factors associated with rates of GWG with P values < 0.2 during bivariate analysis were considered for multivariate analysis using linear regression to determine factors independently associated with rates of GWG. At multivariate analysis, statistical significance was determined if p < 0.05.

## Results

Table 1 summarizes the overall socio-demographic characteristics of the study participants. Two hundred twenty one HIV-negative pregnant women were enrolled into the study. Twenty six were lost to follow up and of the 26, 20 were negative for *H. pylori* infection. However, data from only 192 participants was used to perform all the other analyses because two of the participants lost their pregnancies at 22 and 25 weeks of gestation, one delivered at 35 weeks of gestation. Primigravidae were 68.8% (132/192) while 87.5% (168/192) of the participants were married, 9.9% (19/192) were single and the rest were either divorced/separated or widowed. Only 1.0% (2/192) of the participants were smokers while those who took alcohol were 5.2% (10/192). Nearly two thirds of the participants, 64.6% (124/192), had acquired secondary education. The majority of the participants, 78.1% (144/192), were housewives and only 19.8% (38/192) were employed. Underweight (BMI < 18.5 kg/m^2^) was recorded in 14.6% (28/192) of the participants. Over 99% of the participants were using clean water in their households. We observed no differences in the socio-demographic variables between the participants with *H. pylori* infection and those without *H. pylori* infection. Table 2 shows the means ± standard deviations (sd) and ranges for the selected variables. The mean±sd gestational age at recruitment was 16.9±1.5 weeks with range of 12-18. The mean±sd (range) pre-pregnancy weight, weight at recruitment and age was 53.1±7.6 (37–76) kg, 56.9±8.1 (38-82) kg and 20.9±2.7 (18–35) years respectively. The mean±sd (range) maternal height and maternal pre-pregnancy BMI of participants was 157.4±5.7 (142.0–173.1) cm and 21.3±2.7 (15.0–29.4) kg/m^2^ respectively. The mean rate of weight gain during the second and third trimesters was 300.5±79.7 grams/week with a range of 90–630 grams/week. The mean±sd weight gained by 36 weeks of gestational was 9.6±2.2 kg with a range of 5-16. Table 3 shows the means±sd and ranges for the selected variables and the differences between *H. pylori* positive and *H. pylori* negative participants. The mean pre-pregnancy weight, maternal pre-pregnancy BMI, maternal height and gestation age at delivery of *H. pylori* positive participants were not significantly different from those of *H. pylori* negative participants. Furthermore, the rates of weight gain during second trimester for *H. pylori* negative of 322.3±97.4 grams/week was higher than 294.9±102.4 grams/week for *H. pylori* positive but was not significantly different (P=0.063). However, the mean±sd rate of weight gain during second and third trimesters of 317.0±74.1 grams/week for *H. pylori* negative participants was significantly higher than that of *H. pylori* positive participants (288.2±81.8 grams/week) and significantly different (P = 0.013). The mean±sd gestational weight gained by *H. pylori* negative participants (10.1±2.3kg) by 36 weeks of gestation was higher than that gained by *H. pylori* positive participants (9.2±2.1kg). This difference was significantly different (P = 0.002).

**Table 1:** Socio-demographics characteristics of participants

Variable (N= 192)	Number (%)
**Parity**	
Primigravidae	132 (68.8)
Secundigravidae	60 (31.2)
**Maternal *H. pylori* infection status**	
*H. pylori* positive	110 (57.3)
*H. pylori* negative	82 (42.7)
**Occupation**	
House wife	144 (75)
Peasant	1 (0.5)
Employee	38 (19.8)
Student	9 (2.7)
**Smoking**	
Yes	2 (1.0)
No	190 (99.0)
**Alcohol**	
Yes	10 (5.2)
No	182 (94.8)
**Marital status**	
Married	168 (87.5)
Widowed	2 (1.0)
Divorced/Separated	3 (1.6)
Single	19 (9.9)
**Water source**	
Tap/Borehole	162 (84.4)
Protected well	28 (14.6)
Tank (Harvested rain water)	1 (0.5)
Unprotected well	1 (0.5)
**Building type**	
Permanent	190 (99.0)
Temporary	2 (1.0)
**Household monthly income ($)**	
Low income (<100)	94 (49.0)
Medium income (101-250)	87 (45.3)
High income (> 250)	11 (5.7)
**Education level**	
Low (No education to primary 7)	42 (21.9)
Medium (Secondary level)	124 (64.6)
High (tertiary education)	26 (13.5)
**Maternal pre-pregnancy (BMI kg/m^2^)**	
Underweight (<18.5)	28 (14.6)
Normal weight (18.5-24.9)	143 (74.5)
Overweight (25.0-29.9)	21 (10.9)

**Table 2:** Mean±sd values of some of the participants’ characteristics

Variable	Mean±sd	Min-Max
Age (years)	20.9±2.7	18–35
Gestational age at recruitment (weeks)	16.9±1.5	12–18
Pre-pregnancy weight (kg)	53.1±7.6	37–76
Weight at recruitment (kg)	56.9±8.1	38-82
Maternal pre-pregnancy BMI (kg/m^2^)	21.3±2.7	15.0–29.4
Maternal height (cm)	157.4±5.7	142.0–173.1
Rate of GWG during second trimester (grams/week)	306.6±100.9	80–750
Rate of GWG during third trimester (grams/week)	294.3±101.4	100–600
Average rate of GWG during 2^nd^ & 3^rd^trimesters (grams/week)	300.5±79.7	90–630
Gestational weight gained by 36 weeks of gestation (kg)	9.6±2.2	5.0–16
Gestational age at delivery (weeks)	39.4±1.0	37–42

sd = standard deviation, Min = minimum, Max = maximum

**Table 3:** Mean±sd values selected variables in relation to *H. pylori* infection status

Variable	Mean±sd by H. Pylori		P valve
	*H. pylor*i -ve (n=82)	*H. pylori* +ve (n=110)	
Pre-pregnancy weight (kg)	52.6±7.0	53.5±8.1	0.392
Maternal pre-pregnancy BMI (kg/m^2^)	21.1±2.5	21.6±2.9	0.352
Maternal height (cm)	157.2±5.9	157.5±5.6	0.727
Rate of GWG during second trimester	322.3±97.4	294.9±102.4	0.063
Rate of GWG during third trimester	311.6±103.7	281.4±98.1	0.041
Rate of GWG (second & third trimesters) (grams/week)	317.0±74.1	288.2±81.8	0.013
Gestation weight gained by 36 weeks (kg)	10.1±2.3	9.2±2.1	0.002
Gestation age at delivery	39.4±1.1	39.3±1.0	0.50

sd = standard deviation, n = number, –ve = negative, +ve = positive

### Factors associated with gestational weight gain

For linear regression, the outcome variable was rates of GWG during second and third trimesters. Factors independently associated with GWG were; parity (95% confidence interval (CI) (-0.051 – -0.004; P = 0.023), *H. pylori* infection status (95% CI -0.053 – -0.009; P=0.006), maternal pre-pregnancy BMI (95% CI -0.008 – -0.000; P = 0.037), maternal height (95% CI -0.000 – -0.004; P = 0.022) and household monthly income (-0.023 – -0.005; P = 0.003) (Table 4).

**Table 4:** Factor independently associated with GWG during the second and third trimesters

Variable	Standardized Coefficients (Beta)	*P* valve	95% confidence interval
Parity	-0.158	0.023	-0.051 – -0.004
*H. pylori* infection	-0.192	0.006	-0.053 – -0.009
Maternal pre-pregnancy BMI	-0.142	0.037	-0.008 – -0.000
Maternal height	0.156	0.022	-0.000 – -0.004
Household monthly income	-0.207	0.003	-0.023 – -0.005

## Discussion

The pattern of maternal weight gain during pregnancy is an important determinant of fetal growth [9]. Although several studies have reported how differences in the timing of maternal weight gain may be related to fetal growth outcomes [28, 29], none addresses the effect of maternal *H. pylori* infection on GWG especially in a developing country. In this paper, we investigated the relationship between maternal *H. pylori* infection and rates of GWG during the second and third trimesters of pregnancy because these trimesters greatly influence birth outcome [9]. Although effects of *H. pylori* infection on fetal growth and birth outcome are known [17-20], our study is among the first to evaluate the relationship between *H. pylori* infection and rates of GWG during the second and third trimesters among primigravidae and secundigravidae. We found that the presence of *H. pylori* infection significantly affects the rate of GWG in this population.

In this current study we found the mean±sd rate of GWG of *H. pylori* positive pregnant women (288.2±81.8 grams/week) to be significantly lower than that of *H. pylori* negative pregnant women (317.0±74.1 grams/week), P = 0.013. We did not come across any published information relating *H. pylori* infection to GWG but studies involving non-pregnant have associated *H. pylori* infection with reduced appetite and weight loss [25, 26]. Our study found no differences in pre-pregnancy weight and pre-pregnancy BMI between *H. pylori* positive and *H. pylori* negative participants. This can be explained by the fact that the participants were more of a homogenous and apparently healthy population. This same study also found out that the mean rate of weight gain in primi-gravidae was higher than in secudi-gravidae. This finding is in agreement with recent findings of other studies [30-33] which found that primigravidae are more likely to gain a greater amount of gestational weight and experience excessive GWG than their multigravidae counterparts. This present study further found a positive correlation between rates of GWG during the second and third trimesters and birth weight (P < 0.001). Our finding agrees with several other studies [34-36].

*Helicobacter pylori* infection was found to be independently associated with low rates of gestation weight gain (P = 0.006). Studies have associated *H. pylori* infection with weight loss [25] and weight gain after eradication [21]. One of the mechanisms through *H. pylori* infection may lead to low GWG is by reducing the production of ghrelin and increasing the production of gastric leptin [21, 22]. Ghrelin increases appetite and facilitates fat storage [23] whereas leptin reduces appetite and leads to weight loss [25]. Maternal pre-pregnancy BMI was also found to be independently associated with the rate of GWG (P = 0.037). This is in agreement with other studies that have showed that low pre-pregnancy BMI increases rates of maternal weight gain [37]. Total gestational weight gain and rate of weight gain decreases with increasing pre-pregnancy body mass index [9,14]. Parity was another factor independently associated with rate of GWG (P = 0.023) in this study as seen in Table 4. This finding is in agreement with the findings of other studies which found that primigravidae are more likely to gain a greater amount of gestational weight and experience excessive GWG than their multigravidae counterparts [30-33]. Furthermore, household monthly income was also found to be associated with GWG during the 2^nd^ and 3^rd^ trimesters. In sub Saharan Africa, increased income is associated with lifestyle factors including increased food intake especially calories and reduced physical activity [38]. These factors have been associated with increased total GWG [39, 40]. Although Pickett and colleagues [41] found no interaction between maternal height and net pregnancy weight gain, our present findings show that there is a significant relationship between maternal height and rates of GWG during the second and third trimesters. This is in agreement with the findings of several authors [42, 43]. The strength of our study lies in the fact it was a prospective cohort and we were able to control for some of the known risk factors for GWG such as chronic and genetic diseases. We also included a homogenous population and we are able to attribute the rates of GWG to *H. pylori* infection. However, this current study had some limitations. We did not collect data of all the risk factors for low GWG, for example, level of physical activity during pregnancy, number of antenatal visits, previous poor pregnancy outcome for secundigravidae, neither did we consider other infections, such as malaria and helminth infestations, which are endemic in the study area and have been associated with low rates of GWG [44, 45].

## Conclusion

*Helicobacter pylori* infection has a negative effect on GWG during second and third trimesters. Other factors which independently affect GWG are parity, household monthly income, maternal height and pre-pregnancy BMI. We recommend that women of child bearing age be screened for *H. pylori* infection. Those found positive for *H. pylori* infection should be treated before they become pregnant since drugs used in the treatment of *H. pylori* infection are not safe in pregnancy.

### What is known about this topic

- Parity has an effect on the rate of gestational weight gain;
- Pre-pregnancy body mass index and height also have an an effect on gestational weight gain;
- Level of household income significantly affects the rate of gestational weight gain.

### What this study adds

- Maternal *H. pylori* infection negatively affects the rates of gestational weight gain during the second and third trimesters of pregnancy;
- Maternal *H. pylori* infection has no effect on pre-pregnancy weight and height.

## Competing interests

The authors declare no competing interests.
